# Growth and Physiological Responses and Selection of Tedera (*Bituminaria bituminosa* L.) Genotypes Under Salt Stress Conditions

**DOI:** 10.3390/plants14233618

**Published:** 2025-11-27

**Authors:** Gülcan Kaymak Bayram, Mehmet Can, Utku Tunalı, Zeki Acar, İlknur Ayan

**Affiliations:** 1 Department of Field Crops, Ondokuz Mayıs University, 55100 Samsun, Türkiye; zir.mehmet@gmail.com (M.C.); zekiacar@omu.edu.tr (Z.A.); ilknuray@omu.edu.tr (İ.A.); 2 Field Crops Central Research Institute, 06170 Ankara, Türkiye; utunali1@gmail.com

**Keywords:** tedera, abiotic stress, salinity, proline, mineral matter

## Abstract

Tedera (*Bituminaria bituminosa* L.) is a highly resilient perennial legume that maintains high forage quality under drought and poor fertility conditions with minimal inputs. Identifying salt-tolerant genotypes is crucial for rehabilitating degraded soils and promoting sustainable, low-input production systems. This study evaluated the effects of increasing salinity on the growth and physiological responses of 12 high-yielding tedera genotypes grown under five NaCl concentrations (0, 25, 50, 75, and 100 mM). Growth parameters, photosynthetic pigments, lipid peroxidation, proline amount and mineral substance contents were determined. Increasing NaCl doses caused significant decreases in plant height, leaf number, stem and root weights, while lipid peroxidation and proline accumulation increased. Several genotypes were able to maintain their chlorophyll content and growth performance even under high salinity levels, indicating potential salt tolerance. Correlation analysis indicated a significant negative relationship between the proline concentration and the chlorophyll content. G1, G2, and G3 genotypes showed the highest values when plant height (respectively, 52.23, 52.96 and 52.06 cm), leaf number (53.66, 51.53 and 47.53 per plant), and stem fresh (25.74, 24.56, and 24.44 g) and dry weights (16.78, 16.26 and 15.54 g) were considered together. In the control treatment, the contents of chlorophyll a, chlorophyll b, and carotenoids were 1.362, 0.016, and 0.006 mg g^−1^, respectively, which declined to 0.726, 0.006, and 0.003 mg g^−1^ at 100 mM NaCl. The average proline content increased from 1.743 µmol g^−1^ in the control to 3.403 µmol g^−1^ at 100 mM NaCl dose. When above-ground biomass yield, leaf number, chlorophyll content, proline content, and lipid peroxidase value are considered together, the G1 and G2 tedera genotypes, which are less affected by salinity stress, can be recommended. These findings provide a physiological basis for breeding salt-tolerant tedera cultivars suitable for saline regions of Türkiye.

## 1. Introduction

Soil salinity is one of the most critical challenges limiting agricultural production. Factors such as climate change, increasing drought, and poor irrigation management further exacerbate salinity problems, threatening the productivity of fertile lands [1]. The accumulation of Na^+^, Cl^−^, and other associated ions in the soil beyond normal concentrations disrupts cellular functions in plants, leading to impairments in key metabolic processes such as seed germination and photosynthesis, which may cause severe tissue damage and, under extreme conditions, result in plant death [2]. According to FAO, considering the growing global demand for food, salinity and drought are expected to affect larger areas more severely in the coming years, making salt- and drought-tolerant plants of strategic importance [3]. Tedera (*Bituminaria bituminosa* L.) Stirton genotypes are the most promising new option for drought-prone areas with large ecological adaptation to climatic changes [4]. Ref. [5] reported that tedera seems to be physiologically more adapted to abiotic stress than lucerne (*Medicago sativa* L.), which is quite moderately salt-tolerant. This study aims to determine whether *Tedera*, a species known for its high adaptability to arid environments, also exhibits strong adaptability to saline conditions by examining parameters.

In this context, forage crops play a crucial role not only in providing protein sources for livestock but also in ensuring adaptability to environmental stress conditions. Tedera is one such species with significant potential. Traditionally used as a fodder plant for ruminants in Spain and North African countries, tedera is regarded as an alternative protein source due to its high nutritional value [6]. Recent studies have also demonstrated its positive impact on poultry feeding. Tedera improved the growth performance of chicks, making it a valuable alternative particularly in tropical regions where access to affordable protein sources is limited [7].

Beyond its nutritional qualities, tedera provides significant environmental benefits. Its high tolerance to heat and drought, combined with its ability to maintain growth and green cover throughout the summer, enables it to thrive in marginal areas such as sloped lands, stony soils, shallow soils, or eroded sites without irrigation [8]. Moreover, by utilizing deep underground water more efficiently than other species, tedera helps prevent secondary salinity and reduces the risk of soil erosion. Therefore, due to both its feeding value and its remarkable environmental adaptability, tedera is increasingly recognized as a promising alternative forage crop for sustainable livestock production and agricultural systems.

Various physiological processes occur in plants under salinity stress. One of the primary effects of stress is the degradation of plant cells, which leads to lipid peroxidation [9]. Lipid peroxidation is a prevalent response observed under many types of stress and indicates damage to cell membranes and the disruption of membrane integrity. In addition to lipid peroxidation, plants often develop adaptive mechanisms under salinity stress by accumulating small, water-soluble molecules such as proline within their cells. Numerous studies have demonstrated a positive correlation between the synthesis of organic compounds like proline and increased stress tolerance [10]. Proline is a water-soluble amino acid that accumulates under stress conditions and serves as an indicator of a plant’s tolerance capacity [11]. However, the synthesis of such organic compounds requires a considerable amount of energy. Under salinity stress, the energy demand for the accumulation of both inorganic ions and organic osmolytes increases, thereby reducing the energy available for plant growth. Consequently, this results in reduced biomass accumulation and yield [12]. In summary, plants under stress synthesize proline as a tolerance mechanism and allocate more energy from photosynthesis to proline production, slowing down their growth and development.

One of the most prominent effects of salt stress on plants is the alteration of photosynthetic pigment synthesis. Photosynthesis, which occurs in chloroplasts, is a vital metabolic process for sustaining plant life. Chlorophylls and carotenoids play essential roles in photosynthesis. High salinity disrupts the molecular structure of chlorophylls. Numerous studies have shown that plants respond to salinity by decreasing their photosynthetic rate [13]. Such reduction in photosynthetic activity leads to significant yield losses. Studies have also demonstrated that prolonged exposure to high concentrations of salt results in decreased fresh and dry biomass in both stems and roots [14,15].

Excess sodium (Na^+^) and chloride (Cl^−^) ions in the soil solution reduce the cellular activity of essential nutrients such as calcium (Ca^2+^), potassium (K^+^), and magnesium (Mg^2+^), as well as nitrogen accumulation [16]. This imbalance results in increased ionic and osmotic stress, thereby restricting water and nutrient uptake by plants [17]. The toxicity induced by salinity primarily arises from the excessive accumulation of Na^+^ ions in the cytoplasm, which disrupts intracellular ion homeostasis [18,19]. As a protective mechanism, plant cells attempt to compartmentalize surplus Na^+^ into vacuoles [20]. However, elevated cytoplasmic Na^+^ levels inhibit K^+^ uptake, consequently impairing vital cellular functions. Potassium plays a crucial role in maintaining cell turgor, membrane potential and integrity, and enzyme activation [21]. Most cells maintain a relatively high concentration of K^+^ and a relatively low concentration of Na^+^ in the cytoplasm to ensure their physiological activity. Therefore, a large amount of external Na^+^ influx will hamper K^+^ influx, which leads to plant damage caused by K^+^ deficiency [22]. Similarly, in *Artemisia dracunculus* L., salinity stress has been reported to cause significant decreases in growth parameters, including plant height, leaf number, leaf area, and both the fresh and dry biomass of leaves and stems. In the same study, total protein, chlorophyll, and carotenoid contents declined with increasing NaCl concentrations, while the distribution of nutrient elements such as Mg, Mn, Fe, K, Ca, and Na was substantially altered, indicating disrupted nutrient balance under salt stress [23].

However, little research work has been carried out to study the response of *B. bituminosa* under salinity. This study aims to elucidate the acclimation mechanisms of different tedera genotypes grown under various salt concentrations and to provide insights into their potential use in saline agricultural systems. In this study, eleven local genotypes and one genotype originally from Spain, selected for their greater yield and agronomic qualities, were used. Furthermore, the connections between the studied genotypes’ mineral nutrient use and salt concentration are evaluated for the first time. These aspects make our research original and creative.

## 2. Results

As the average of salt doses, the tedera genotypes examined in the study exhibited statistically significant differences in plant height, leaf number, and stem and root bio-mass. For each of these traits, significant variations between the genotypes were found, and these showed that G1, G2, and G3 genotypes showed the highest values when plant height, leaf number, and stem fresh and dry weights were considered together. The other genotypes, on the other hand, showed higher values in terms of root fresh and dry weights. Increasing salt doses had adverse effects on both leaf number and stem dry weight. In particular, leaf number responded more sensitively to elevated NaCl doses, showing a significant decrease as stress intensity increased. While the fresh weight of the roots advanced, the dry weight of the stems decreased as the concentration of salt increased (Table 1).

Averaged across the genotypes, dry stem weights decreased with increasing salt doses. At low doses (especially 25 mM), a partial increase in fresh stem weight was observed; however, this trend reversed at higher doses, leading to a marked reduction in above-ground biomass production. Conversely, increasing salt doses resulted in higher root fresh weight.

Root fresh and dry weights generally had an increasing trend under salt stress (100 mM). This may suggest that the plant develops morphological adaptations by enhancing its root system to improve water and nutrient uptake. Additionally, the fact that differences among genotypes were statistically significant for most parameters indicates that some varieties were able to maintain higher root biomass even under salt stress conditions. Still, at doses exceeding a certain threshold, plant growth is significantly inhibited. On the other hand, there was no statistically significant impact of increasing salt dosages on plant height, stem fresh weight, or stem dry weight. For height of plants and leaf number, the genotype-salt dose interaction was shown to be highly significant (Table 1).

According to the results of variance analysis, significant differences were observed among tedera genotypes in terms of calcium content. While some genotypes stood out with high calcium accumulation, others had lower levels. Statistically significant differences were also found among NaCl doses regarding calcium content. A notable increase in calcium levels was observed with increasing NaCl doses (except 50 mM dose), indicating the role of calcium in plant defense under stress conditions.

Significant differences were also detected among genotypes in terms of magnesium content. Some genotypes exhibited high magnesium accumulation, while others had lower values. It is thought that genotypes with higher magnesium content may better preserve their photosynthetic systems under stress due to the critical role of magnesium in photosynthesis. Although the applied NaCl doses caused a general change in magnesium content, this change was not statistically significant.

Regarding potassium content, statistically significant differences were found among both genotypes and salt treatments. While some genotypes accumulated higher levels of potassium, others had lower potassium content. In terms of NaCl treatments, an increase in potassium content was observed at lower salt doses, but potassium levels declined as salt dose increased. When Ca, Mg, and K contents were considered together, the G3, G11, and G12 genotypes were prominent, and no significant genotype-dose interaction was found for any of the three mineral substances (Table 2).

The analysis of variance also indicated that chlorophyll a, chlorophyll b, and carotenoid contents were significantly affected by NaCl doses. A general decreasing trend was observed in the amounts of chlorophyll a, chlorophyll b, and carotenoids as salinity increased.

**Figure 1:**
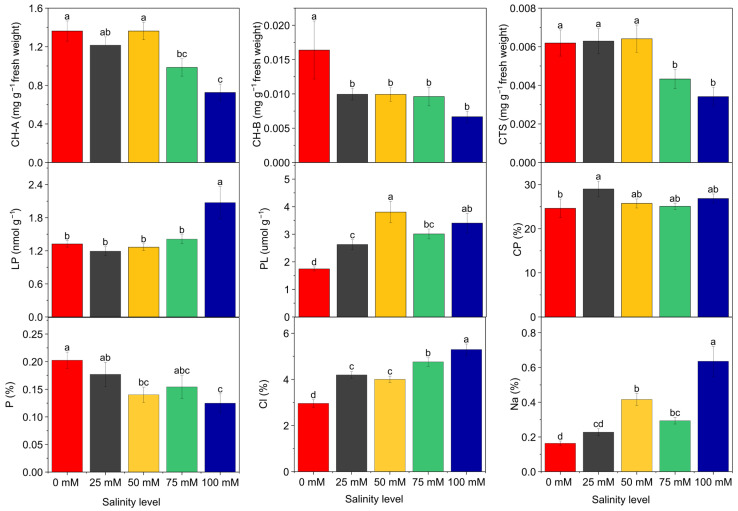
Physiological and nutritional parameters determined in Tedera genotypes grown under different NaCl concentrations (0, 25, 50, 75, and 100 mM). The figure shows changes in photosynthetic pigments, proline accumulation, and mineral nutrient (P, Na, Cl) contents. Bars represent mean ± SE, and different letters indicate significant differences according to Duncan’s test (*p* ≤ 0.05).

In the control treatment, the contents of chlorophyll a, chlorophyll b, and carotenoids were 1.362, 0.016, and 0.006 mg g^−1^, respectively, which declined to 0.726, 0.006, and 0.003 mg g^−1^ at 100 mM NaCl. These results suggest that salt stress suppresses photosynthetic pigments, potentially reducing the plant’s photosynthetic capacity (Figure 1).

Analysis of variance revealed significant differences among NaCl doses in terms of lipid peroxidation levels. The average lipid peroxidation, measured as malondialdehyde (MDA), was 1.323 nmol MDA g^−1^ in the control group, which increased to 2.073 nmol MDA g^−1^ at 100 mM NaCl dose. Similarly, proline content showed statistically significant differences between NaCl doses. The average proline content increased from 1.743 µmol g^−1^ in the control to 3.403 µmol g^−1^ at 100 mM NaCl dose (Figure 1).

Regarding crude protein content, significant differences were also observed among NaCl treatments. On average implementation of salt doses increased crude protein con-tent. The highest crude protein content was recorded at 25 mM NaCl (29.00%), while it was 24.64% in the control. At 100 mM NaCl, the crude protein content was 26.84% (Figure 1). In addition, phosphorus content varied significantly with NaCl treatments as well. The highest phosphorus content (0.20%) was found in the control treatment, and a decline was observed as salinity levels increased. This suggests that elevated salinity may restrict phosphorus uptake or utilization (Figure 1).

According to the results of variance analysis, there was a statistically significant difference between NaCl doses in terms of sodium and chloride content. When the chloride ratio was examined, the average value of 2.96% in the control group increased at 25 mM (4.19%), 50 mM (4.01%), 75 mM (4.75%) and 100 mM (5.29%) levels. It was determined that the sodium content, which was an average of 0.16% in the control dose, increased by 0.23% at 25 mM, 0.42% at 50 mM, 0.29% at 75 mM and 0.64% at 100 mM (Figure 1).

**Figure 2:**
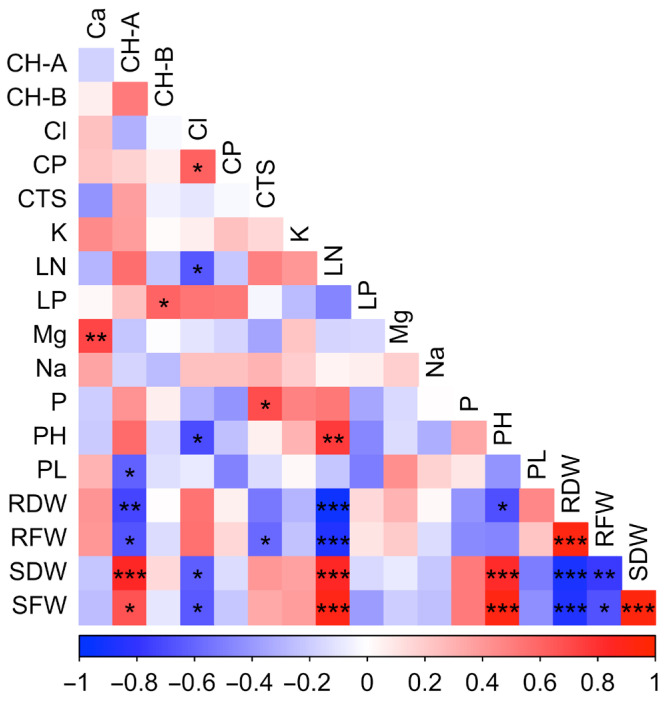
Correlation heat map showing the relationships among growth, physiological, biochemical, and mineral parameters measured in tedera under salt stress. Positive correlations are shown in red and negative correlations in blue, with color intensity indicating correlation strength. Asterisks denote significance levels (* *p* ≤ 0.05, ** *p* ≤ 0.01, *** *p* ≤ 0.001).

The correlation table shows the relationships between the investigated traits (Figure 2). Statistically significant (*p* ≤ 0.001) and positive correlations were found between plant height (PH), stem fresh weight (SFW) and stem dry weight (SDW). This situation shows that the parameters related to vegetative growth have a structure that supports the development of each other. The statistically significant negative relationship between chloride (Cl^−^) and stem fresh (SFW) and dry weight (SDW) indicates that increasing salt dose negatively affects biomass production. This situation is consistent with the literature information that excessive accumulation of Cl^−^ ions causes osmotic stress, ion imbalance and metabolic disorders in the plant and slows down the growth processes.

The negative and significant correlation (*p* ≤ 0.05) between proline and chlorophyll a (CH-A) show that proline accumulation increases under stress conditions while chlorophyll levels decrease. This condition shows that stress-induced proline synthesis, as part of the plant’s defense mechanisms, may lead to the accumulation of reactive oxygen species (ROS), triggering chlorophyll degradation. Thus, despite the protective role of proline in osmotic balance under stress conditions, chlorophyll loss causes a decrease in photosynthetic capacity and shows that it reduces the growth potential of the plant. The negative correlation between proline and aboveground biomass further supports this conclusion.

Correlations between macro and micronutrient elements provide important findings in terms of evaluating the effect of salt stress on ion balance. The positive correlation between calcium (Ca) and magnesium (Mg) suggests that these elements may jointly contribute to maintaining cellular homeostasis and stabilizing plant cell membranes under salt stress conditions.

**Figure 3:**
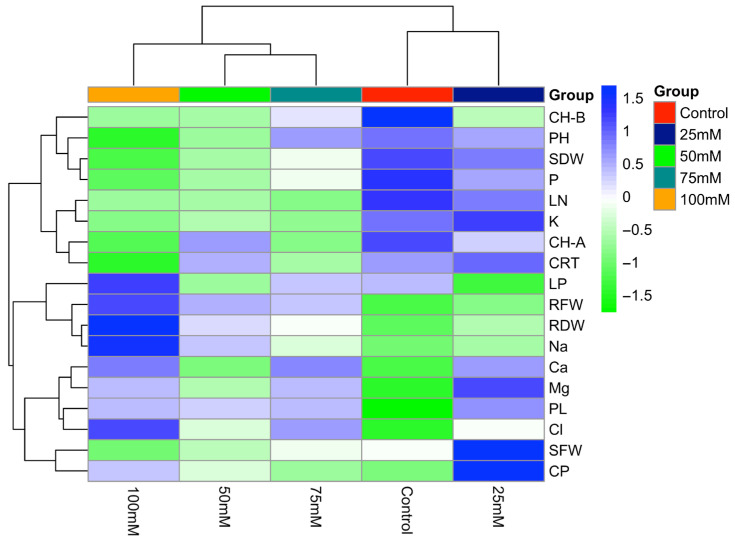
Heat map illustrating the effects of different NaCl concentrations (0, 25, 50, 75, and 100 mM) on growth, physiological, and biochemical traits of tedera.

The presented heat map illustrates the effects of different salinity levels on the studied traits (Figure 3). Generally, increasing salt dose resulted in a decline in the value of leaf number, stem dry weight, chlorophyll b content, potassium, and phosphorus. Chlorophyll b reached its highest level under the control treatment, because higher NaCl doses likely suppressed its synthesis. Conversely, lipid peroxidation, root fresh and dry weights, sodium, and chloride contents increased with rising salinity doses. Additionally, proline content was lowest in the control group but increased progressively with salt dose, supporting the role of osmotic adjustment in the plant’s stress adaptation process. At the highest salinity dose (100 mM NaCl), a marked decrease was observed in growth-related parameters (plant height, stem fresh weight, stem dry weight, leaf number), certain physiological traits (chlorophyll a and b, carotenoid contents), and mineral nutrients (phosphorus and potassium). In addition, the decrease in potassium and increase in sodium content with increasing salt dose may have caused an ion imbalance. As a result, salt stress shows that it negatively affects the growth and physiological development of the plants.

**Figure 4:**
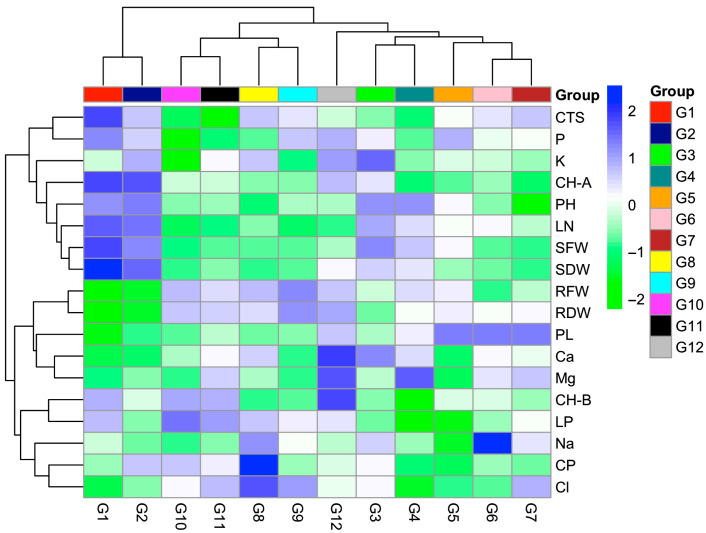
Heat map showing the variation in growth, physiological, and biochemical traits among 12 tedera genotypes under salt stress.

When the heat map illustrating the distribution of traits among genotypes was examined, significant differences were observed between genotypes in terms of salt tolerance (Figure 4). Some genotypes (e.g., G1, G2, G12) maintained relatively high chlorophyll content even at high salinity doses. Genotypes G5, G6, and G7 exhibited high proline accumulation alongside low lipid peroxidation levels. The high proline content indicates that these genotypes are more effective in coping with osmotic stress. In addition, these genotypes were able to maintain the carotenoid amount. However, genotype G6 could not maintain ion balance; the sodium content was high, and the potassium content was lower. On the other hand, some genotypes (e.g., G10 and G12) displayed greater sensitivity to salt stress. In terms of proline accumulation of genotypes, tolerant genotypes showed higher levels, highlighting the effectiveness of osmotic adjustment mechanisms.

## 3. Discussion

In the current study, the physiological and agronomical responses of tedera genotypes to different NaCl doses were investigated. The results indicate that salt stress has a significant impact on plant growth and development. Increasing NaCl doses caused a marked reduction in leaf number, stem dry weight, root fresh weight an increase was observed. Decrease in plant height and leaf number can be attributed to the inhibitory effects of salt stress on cell division and elongation. Similarly, [4] found that rising salt concentrations decreased dry biomass yield by 67% and leaf number by 51%. Root wet weight increased with increasing salt concentrations, while root dry weight showed no significant change. Possibly, under salt stress, roots continuously grow to reach water, and these new tissues have a higher water content. This situation caused an increase in root fresh weight [24]. While salt concentration did not significantly alter plant height, the reason for the significant decline in leaf number is that plants transport toxic substances such as sodium chloride to the mesophyll tissues of older leaves to preserve young tissues, resulting in the death of older leaves [4].

This suggests that salt stress slows down growth processes by inducing osmotic and ionic imbalances. Like many other abiotic stresses, salinity stress inhibits plant growth, with the extent of growth reduction depending on various factors such as plant species, developmental stage, and salt concentration [25]. Genotypes responded differently to salt doses regarding plant height, number of leaves, and stem weight. The G1, G2, and G3 genotypes had higher values for these three traits under salt stress. Higher values were obtained for age and dry root weight from other genotypes. Genotypes G1, G2, and G3 may have a better ability to absorb water under high salt concentrations. Since the genotypes were collected from different environments, their responses to stress conditions also differ [4] conducted a study on different local tedera genotypes and determined that the genotypes responded differently to salt stress.

Reduced growth serves as an adaptive strategy that enables plants to withstand and survive under saline conditions. Previous studies have similarly shown that high salinity limits plant growth and reduces biomass production [11,12,24,26,27].

Soil or irrigation water salinity has a detrimental effect on plant development and productivity. While excessive salts in the soil can be removed through specific agricultural practices, such measures do not always yield successful results. In terms of both soil health and more efficient use of marginal areas, genetically salt-resistant plants should be identified and grown in salt-affected areas. Plants exhibit varying levels of salt tolerance not only between species but also among genotypes within the same species [28].

Plant reactions to salinity stress are complex. Salt stress can harm plants through osmotic imbalances, ion toxicity, nutrient uptake disorders, hormonal irregularities, and disruptions to the antioxidant system [29,30]. One of the most visibly affected organs in plants under salt stress is the leaf. NaCl has particularly detrimental effects on chlorophyll content in the leaves. Due to salt stress, photosynthetic pigments (chlorophyll a, chlorophyll b, and carotenoids) decreased significantly. This decline indicates damage to the chloroplast structure and a reduction in photosynthetic capacity [31]. The high concentration of Na^+^ in the soil leads to the accumulation of Na^+^ in plants. Na^+^ of a high concentration will reduce the membrane potential and promote the absorption of Cl^−^ under a chemical gradient. Excessive Na^+^ is harmful to cell metabolism and some enzymes [28]. A high concentration of Na^+^ leads to osmotic imbalance, membrane disfunction, increased production of ROS, and thus affects cell division and growth [29].

There are different values for chlorophyll content among genotypes. Chlorophyll a was found to be greater in G1 and G2 genotypes, while chlorophyll b was higher in G12 genotype compared to other genotypes (Figure 4). According to the results, the G1 and G2 genotypes showed the highest values in terms of leaf number and stem yield. This can be interpreted as these genotypes continuing photosynthesis under high salt concentrations. Chlorophyll content is a fundamental physiological trait that plays a crucial role in plant health and development. While low salinity may increase chlorophyll content, high salinity can disrupt the molecular structure of chlorophyll [32]. Under salt stress, chlorophyll and carotenoid levels in maize and chlorophyll content in some cotton cultivars have been reported to decrease [33]. A decline in chlorophyll content under high salinity (200 mM) in cowpea and jack bean was also reported [34]. The photosynthetic process in plants is negatively affected by salinity stress [29]. Similar results were obtained in this study, where increasing NaCl doses were found to negatively affect chlorophyll content. A significant reduction in chlorophyll content was observed with rising NaCl doses, which is consistent with findings reported in the literature. The decline in chlorophyll content may result from a decrease in chlorophyll synthesis or the degradation of chlorophyll pigments.

Another important finding was the significant increase in proline accumulation with increasing NaCl doses. Proline functions as an osmotic regulator and cellular protectant in plants and typically accumulates under stress conditions [10]. Numerous studies have reported a positive correlation between the synthesis of organic compounds such as glycine betaine and proline and plant stress tolerance [10]. Previous research has demonstrated that proline accumulation is a key component of salt stress tolerance mechanisms [35,36]. It has been reported that proline supports plant survival under stress conditions (salt and drought stress) by protecting cellular enzymes [37]. At the 100 mM NaCl dose (Figure 1), where lipid peroxidation reached its highest level, considerable membrane damage occurred in the plants. Consistently, both leaf number and stem biomass were lowest at this salt concentration, indicating a strong adverse effect on plant growth. This pattern is in agreement with previous reports [10], which similarly documented increased membrane injury and growth reduction under high salinity conditions. In this study, genotypes exhibiting higher proline levels were observed to be more tolerant to salt stress. Heat map analysis revealed that genotypes G5, G6, and G7 showed high proline accumulation, while also exhibiting low levels of lipid peroxidation. Together with a lower proline content than other genotypes and high yields, the proline content of G1 and G2 genotypes can be explained by the fact that these genotypes do not need to synthesize much proline to balance osmotic pressure, even at high salt concentrations. Reference [4], working with different tedera genotypes, reported a negative relationship between proline content and yield. As NaCl doses increased, a decreasing trend in chlorophyll content was observed. However, according to the heat map, genotypes G1, G2, and G12 maintained their chlorophyll content better than the other genotypes. This condition indicates that chlorophyll degradation was less pronounced in these genotypes under stress conditions, allowing them to sustain their photosynthetic capacity more effectively. A similar increase in leaf proline accumulation in the tedera plant subjected to drought stress was reported [5]. Similar results were obtained in this study, exposed to salinity stress. Several researchers have also determined that proline content increases in plants under stress conditions [24,38,39].

When mineral substances were examined, it was determined that sodium (Na^+^) and chloride (Cl^−^) contents increased with increasing NaCl dose, while potassium (K^+^) and phosphorus (P) content decreased. This situation shows that Na^+^ ions prevent K^+^ uptake and create an ion imbalance. Previous studies have similarly shown that salt stress disrupts ion homeostasis and reduces potassium uptake, leading to metabolic disturbances in plants [40]. As salt doses increased, Ca and Mg content in plant tissues also increased, but the increase in Mg content was not found to be significant. This increase was likely due to the rise in soil pH. Indeed, when soil pH increases with rising salt concentration, the availability of Ca and Mg in the soil also increases [41]. This condition indicates that salt stress can affect the uptake and transport of mineral nutrients [17].

These findings clearly demonstrate the suppressive effects of salt stress on growth and physiological parameters in tedera genotypes. Effects of increasing NaCl doses on photosynthetic pigments, mineral balance, and osmotic regulation mechanisms are similar to studies reported in the literature [17,27]. Especially, physiological responses such as proline accumulation and the capacity to preserve chlorophyll content varied among genotypes. These differences suggest that some genotypes may possess greater tolerance to salt stress and should be further investigated for their stress-resistance characteristics. In future research, exploring the molecular mechanisms in tedera genotypes and incorporating them into breeding programs could contribute to the development of salt-tolerant plant cultivars.

It has been determined that the G1 and G2 genotypes are less impacted by salinity stress than other genotypes, especially when the high above-ground biomass yields, high leaf numbers, high chlorophyll contents, low proline content, and moderate lipid peroxidase values are taken into account collectively.

## 4. Materials and Methods

### 4.1. Plant Material and Experimental Area

In this study, 12 Tedera genotypes, previously identified as high-yielding in earlier research, were used. One of the genotypes originated from Spain, while the others were collected from the provinces of Samsun, Sinop, and Kastamonu in Türkiye (Table 3).

The study was conducted in the laboratories and greenhouses of the Faculty of Agriculture at Ondokuz Mayıs University. Each genotype was grown in pots under five different NaCl doses (0, 25, 50, 75, and 100 mM), as three replications. Since tedera seeds have hard seed characteristics, the seeds were first germinated in viols. After reaching the three-leaf stage, the seedlings were transplanted into pots with a depth and diameter of 20 cm. Each pot was uniformly filled with a 3 kg mixture of soil:fertilizer at a ratio of 2:1. The fertilizer was farmyard manure with a pH of 7.4, an EC of 0.76 mmhos/cm, and an organic matter content of 70%. The pH of the soil used in the pots was determined as 7.0, with an electrical conductivity (EC) of 3.053 mmhos/cm and an organic matter content of 2.71%. The fertilizer used had a pH of 7.4, an organic matter content of 70%, and an EC value of 0.76 mmhos/cm. For each dose, solutions were made by mixing the exact amount of NaCl with pure water. The application was carried out at a rate of 200 mL per pot per day. To guarantee that each pot received an equal amount of light, they were relocated at regular intervals.

Initially, plants were irrigated with tap water. Samples were collected daily at 12:00 p.m. from the leaf tissues of three plants per replicate after 21 days of salt stress treatment to ensure consistent environmental and light conditions. The following characteristics were examined in the harvested seedlings.

### 4.2. Plant Growth Measurements

At the end of the 21-day growth period, plant height was measured using a ruler before harvesting. After harvesting, stems and roots were separated. Fresh weights of both stems and roots were recorded, and samples were then dried at 60 °C for 48 h in an oven to determine dry weights.

### 4.3. Photosynthetic Pigments

The chlorophyll a, chlorophyll b, and carotenoid contents of the leaves were determined according to the method described in [42]. For this, 0.1 g of fresh plant tissue was ground in a mortar with 1–2 mL of 80% acetone. The extract was filtered through filter paper, and the final volume was adjusted to 10 mL with 80% acetone. Absorbance values were read using a UV-VIS spectrophotometer at wavelengths of 663 nm (chlorophyll a), 645 nm (chlorophyll b), and 440 nm (carotenoids), using 80% acetone as a blank. Pigment concentrations were calculated using the following formulas based on the absorbance readings:

Chlorophyll a (mg/g) = [12.7 (D663) − 2.69 (D645)] × 10/1000× g fresh weight

Chlorophyll b (mg/g) = [22.9 (D645) − 4.68 (D663)] × 10/1000× g fresh weight

Carotenoid (mg/g) = [4.69 (D440) − (chlorophyll a + chlorophyll b) × 0.286] × 10/1000× g fresh weight

### 4.4. Lipid Peroxidation (MDA Content)

Lipid peroxidation was determined by measuring the malondialdehyde (MDA) content, an end product of membrane lipid peroxidation, according to the method of [43]. The principle of the assay is based on the spectrophotometric detection of the pink-red chromogen formed by the reaction of MDA with thiobarbituric acid (TBA) at high temperature (95 °C), which absorbs at 532 nm. Approximately 0.2 g of fresh leaf tissue was homogenized with glass powder in 2 mL of 10% trichloroacetic acid (TCA) containing 0.25% TBA. The homogenate was incubated at 95 °C for 30 min, rapidly cooled in an ice bath, and centrifuged at 15,000× *g* for 15 min. The absorbance of the supernatant was read at 532 nm. Lipid peroxidation products were expressed as nmol g^−1^ fresh weight using a regression equation.

### 4.5. Proline Content

Proline content was determined according to the method described by [44]. Fresh leaf tissue (0.2 g) was homogenized with glass powder in 4 mL of 3% sulfosalicylic acid and filtered through two layers of glass wool. One milliliter of the filtrate was mixed with 1 mL of acid ninhydrin and 1 mL of glacial acetic acid in a test tube. The mixture was incubated in a water bath at 100 °C for 1 h. After incubation, the tubes were transferred to an ice bath to stop the reaction. Absorbance was read at 546 nm. Proline content was calculated and expressed as µmol g^−1^ fresh weight.

### 4.6. Crude Protein Analysis

Crude Protein Analysis: Crude protein content was determined using the Kjeldahl method. Plant samples were burned with concentrated sulfuric acid (H_2_SO_4_), converting nitrogen (N) into ammonium sulfate ((NH_4_)_2_SO_4_), which was then distilled into ammonium borate. The amount of nitrogen was determined by titration with 0.1 N hydrochloric acid (HCl). Crude protein content was calculated by multiplying the total nitrogen content by the factor 6.25.

### 4.7. Determination of Na, K, Ca, and Mg Content

Plant samples were subjected to dry ashing. The resulting ash extract was analyzed using an Atomic Absorption Spectrophotometer to determine sodium (Na), potassium (K), calcium (Ca), and magnesium (Mg) concentrations.

### 4.8. Total Phosphorus

Total P content was determined with a spectrophotometer from the dry-ashed plant extract [45].

### 4.9. Chlorine Determination

Cl content was measured using the Mohr method following the procedure described in [46]. Chlorine was determined by titrating with AgNO_3_ using a potassium chromate indicator.

#### Statistical Analysis

The data obtained were analyzed using the SPSS 25.0 statistical software package. Before performing ANOVA, the data were tested for normality using the Shapiro–Wilk test and for homogeneity of variances using Levene’s test. After confirming these assumptions, one-way ANOVA was conducted. Statistical differences among treatment means were determined using Duncan’s multiple range test. Hierarchical heat map and correlation analyses were conducted using the RStudio software (Version 2025.09.2+418) environment (https://posit.co/products/open-source/rstudio/ (accessed on 6 September 2025). The corrplot package was used for correlation analysis, while the heatmap package was employed for heat map visualizations.

## 5. Conclusions

The findings of this study provide valuable insights into the definition of salt-tolerant genotypes and the selection of potential candidates for breeding programs aimed at enhancing salt stress resilience. When above-ground biomass yield, leaf number, chlorophyll content, proline content, and lipid peroxidase value are considered together, the G1 and G2 Tedera genotypes, which are less affected by salinity stress, can be recommended. These findings provide a physiological basis for breeding salt-tolerant Tedera cultivars suitable for saline regions of Türkiye.

Future studies focusing on the genetic and molecular pathways regulating these tolerance mechanisms may contribute to the development of effective strategies for improving salt tolerance in plants. In the long term, the utilization of salt-tolerant genotypes and the implementation of sustainable agricultural practices will play a crucial role in mitigating the adverse effects of salinity stress.

## Figures and Tables

**Table 1:** Growth characteristics determined in tedera.

Genotypes	Plant	Leaf	Stem Fresh	Stem Dry	Root Fresh	Root Dry
	Height (cm)	Number	Weight (g)	Weight (g)	Weight (g)	Weight (g)
G1	52.23 ± 9.04 a	53.66 ± 15.75 a	25.74 ± 4.27 a	16.78 ± 3.46 a	16.34 ± 0.90 d	13.50 ± 0.32 d
G2	52.96 ± 11.09 a	51.53 ± 15.25 ab	24.56 ± 3.81 ab	16.26 ± 1.08 ab	16.73 ± 1.61 cd	13.68 ± 0.57 cd
G3	52.06 ± 8.30 a	47.53 ± 7.44 abc	24.44 ± 2.98 ab	15.54 ± 0.84 abc	17.65 ± 2.13 abc	13.94 ± 0.81 bc
G4	52.30 ± 12.82 a	42.93 ± 9.28 bcd	23.39 ± 1.96 bc	15.40 ± 0.55 bc	18.10 ± 1.70 ab	14.13 ± 0.75 ab
G5	47.16 ± 8.34 ab	39.86 ± 9.39 cde	22.38 ± 3.77 bcd	14.78 ± 3.16 c	17.92 ± 1.30 abc	14.18 ± 0.64 ab
G6	43.10 ± 13.43 ab	40.60 ± 10.36 cde	20.49 ±2.40 d	14.64 ± 0.62 c	17.19 ± 1.59 bcd	14.13 ± 0.56 ab
G7	36.86 ± 13.99 c	36.26 ± 9.27 def	20.25 ± 1.69 d	14.46 ± 0.43 c	17.58 ± 1.43 abc	14.17 ± 0.49 ab
G8	40.46 ±12.97 ab	33.26 ± 9.89 def	20.44 ± 2.10 d	14.48 ± 0.55 c	18.28 ± 2.23 ab	14.24 ± 0.71 ab
G9	43.96 ± 7.63 ab	28.53 ± 8.08 f	20.60 ± 2.62 d	14.50 ± 0.61 c	18.60 ± 4.10 a	14.42 ± 2.77 a
G10	42.73 ± 15.46 ab	27.13 ± 8.27 f	20.18 ± 1.34 d	14.48 ± 0.86 c	18.27 ± 1.02 ab	14.30 ± 0.52 ab
G11	43.26 ± 13.31 ab	29.60 ± 10.41 f	20.44 ± 1.54 d	14.69 ± 0.44 c	18.06 ± 1.85 ab	14.26 ± 0.72 ab
G12	43.96 ± 10.25 ab	30.93 ± 7.21 ef	21.25 ± 2.21 cd	15.28 ± 0.69 bc	18.19 ± 1.25 ab	14.37 ± 0.49 ab
Dose						
0 mM	50.31 ± 10.59	39.81 ± 15.31 a	21.46 ± 3.98	15.72 ± 3.13 a	17.21 ± 0.92 b	14.04 ± 0.45
25 mM	45.19 ± 12.63	34.72 ± 13.28 ab	22.28 ± 3.87	15.00 ± 1.04 ab	18.30 ± 3.06 a	14.60 ± 1.89
50 mM	42.77 ± 12.89	30.91 ± 10.04 b	21.17 ± 3.09	14.76 ± 0.75 ab	18.28 ± 1.71 a	14.25 ± 0.60
75 mM	41.58 ± 14.86	30.33 ± 10.67 b	21.13 ± 2.87	14.96 ± 0.84 ab	18.49 ± 1.59 a	14.38 ± 0.62
100 mM	41.66 ± 13.96	28.38 ± 12.47 b	19.97 ± 2.61	14.21 ± 0.86 b	19.01 ± 2.17 a	14.59 ± 0.91
Genotype	**	**	*	*	*	**
NaCl	ns	*	ns	*	*	ns
GxD	**	**	ns	ns	ns	ns

Data are presented as the mean ± standard deviation (SD). Different letters indicate significant differences at * *p* ≤ 0.05, ** *p* ≤ 0.01 level within one parameter. (ns) non-significant.

**Table 2:** Some nutrients identified in tedera.

Genotypes	Calcium (%)	Magnesium (%)	Potassium (%)
G1	1.19 ± 0.282 c	0.27 ± 0.05 b	0.30 ± 0.07 bcd
G2	1.24 ± 0.39 c	0.31 ± 0.05 b	0.35 ± 0.05 abc
G3	1.91 ± 1.34 ab	0.34 ± 0.02 ab	0.37 ± 0.09 a
G4	1.70 ± 0.55 abc	0.54 ± 0.35 a	0.28 ± 0.07 cd
G5	1.23 ± 0.22 c	0.24 ± 0.05 b	0.30 ± 0.03 bcd
G6	1.62 ± 0.50 abc	0.40 ± 0.27 ab	0.30 ± 0.04 bcd
G7	1.56 ± 0.54 abc	0.44 ± 0.26 ab	0.29 ± 0.05 bcd
G8	1.71 ± 0.41 abc	0.32 ± 0.04 b	0.34 ± 0.07 abc
G9	1.33 ± 0.29 bc	0.28 ± 0.03 b	0.26 ± 0.06 de
G10	1.46 ± 0.65 bc	0.28 ± 0.10 b	0.21 ± 0.10 e
G11	1.61 ± 0.43 abc	0.42 ± 0.18 ab	0.32 ± 0.07 a–d
G12	2.08 ± 0.51 a	0.54 ± 0.21 a	0.36 ± 0.05 ab
Dose			
0 mM	1.17 ± 0.21 b	0.29 ± 0.05	0.34 ± 0.05 a
25 mM	1.73 ± 0.90 a	0.43 ± 0.25	0.36 ± 0.08 a
50 mM	1.28 ± 0.36 b	0.34 ± 0.19	0.28 ± 0.06 b
75 mM	1.78 ± 0.57 a	0.39 ± 0.15	0.27 ± 0.08 b
100 mM	1.79 ± 0.53 a	0.39 ± 0.23	0.27 ± 0.08 b
Genotype	*	*	*
NaCl	**	ns	**
GxD	ns	ns	ns

Data are presented as the mean ± standard deviation (SD). Different letters indicate significant differences at * *p* ≤ 0.05, ** *p* ≤ 0.01 level within one parameter. (ns) non-significant.

**Table 3:** List of tedera genotypes used in the study.

Genotype Number	Collection Site	Coordinates	
G1	Spain	-	-
G2	Kastamonu İnebolu	41°58′32.8″	33°46′10.4′
G3	Kastamonu-Çatalzeytin	41°57′48.4″	34°09′07.8′
G4	Sinop Kanlıçay	41°40′40.3″	35°22′22.8′
G5	Samsun-Kozağzı	41°28′05.1″	35°49′56.8′
G6	Samsun-Çarşamba	41°04′35.1″	36°40′09.0′
G7	Samsun-Bağkur	41°18′39.0″	36°20′02.5′
G8	Samsun-Baruthane	41°19′08.5″	36°19′13.6′
G9	Samsun-Nebyan	41°23′35.9″	35°59′06.2′
G10	Samsun-Kurupelit	41°22′16.0″	36°11′46.7′
G11	Sinop-Tıngıroğlu	41°47′41″	35°00′23″
G12	Samsun-Kavak	41°03′14.35″	35°56′59.84″

## Data Availability

The data supporting the results of this study are available from the corresponding author upon reasonable request. The data are not publicly available due to institutional policy and project confidentiality.

## Abbreviations

The following abbreviations are used in this manuscript:

PH	Plant height
SFW	Stem fresh weight
SDW	Stem dry weight
RFW	Root fresh weight
RDW	Root dry weight
CH-A	Chlorophyll a
CH-B	Chlorophyll b
CTS	Carotenoids
LP	Lipid peroxidation
PL	Proline
CP	Crude protein
P	Phosphorus content
Cl	Chlorine content
Na	Sodium content
Mg	Magnesium content
Ca	Calcium content
K	Potassium content
ROS	Reactive oxygen species
